# Impacts of Selective Predation on Infection Prevalence and Host Susceptibility

**DOI:** 10.1002/ece3.70778

**Published:** 2025-01-21

**Authors:** Stephanie O. Gutierrez, Ximena E. Bernal, Catherine L. Searle

**Affiliations:** ^1^ Department of Biological Sciences Purdue University West Lafayette Indiana USA; ^2^ Department of Biology Emory University Atlanta Georgia USA; ^3^ Smithsonian Tropical Research Institute Ancón Republic of Panama

**Keywords:** *Daphnia*, evolution, *Metschnikowia*, parasite, pathogen, selection

## Abstract

Predation can alter diverse ecological processes, including host–parasite interactions. Selective predation, whereby predators preferentially feed on certain prey types, can affect prey density and selective pressures. Studies on selective predation in infected populations have primarily focused on predators preferentially feeding on infected prey. However, there is substantial evidence that some predators preferentially consume uninfected individuals. Such different strategies of prey selectivity likely modulate host–parasite interactions, changing the fitness payoffs both for hosts and their parasites. Here we investigated the effects of different types of selective predation on infection dynamics and host evolution. We used a host–parasite system in the laboratory (*Daphnia dentifera* infected with the horizontally transmitted fungus, *Metschnikowia bicuspidata* ) to artificially manipulate selective predation by removing infected, uninfected, or randomly selected prey over approximately 8–9 overlapping generations. We collected weekly data on population demographics and host infection and measured susceptibility from a subset of the remaining hosts in each population at the end of the experiment. After 6 weeks of selective predation pressure, we found no differences in host abundance or infection prevalence across predation treatments. Counterintuitively, populations with selective predation on infected individuals had a higher abundance of infected individuals than populations where either uninfected or randomly selected individuals were removed. Additionally, populations with selective predation for uninfected individuals had a higher proportion of individuals infected after a standardized exposure to the parasite than individuals from the two other predation treatments. These results suggest that selective predation can alter the abundance of infected hosts and host evolution.

## Graphical abstract

We tested the impact of selective predation on populations of infected hosts. We found that selective predation for infected hosts had higher abundance of infected individuals, while selective predation for uninfected individuals resulted in higher susceptibility to the parasite.

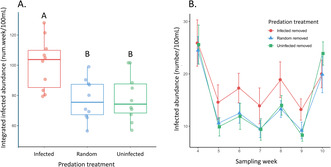

## 1 Introduction

Host–parasite interactions influence and are influenced by connections with other community members, including predators. The direct effects of predators on their prey can produce cascading effects on prey disease dynamics through various direct and indirect mechanisms (Bruno and Cardinale 2008). For example, the presence of predators often reduces overall prey densities and can also cause changes to prey phenotype and behavior, which can impact host susceptibility and exposure to parasites (Hamilton and Zuk 1982; Dobson 1988). Additionally, predators are often selective in the prey they consume, exerting stronger predation on some classes of the population than others, which has the potential to impact host demographics and subsequently disease dynamics.

Predators can select prey based on a variety of features, including infection status, which may have indirect and complex effects on their prey. For example, it is common for predators to preferentially select prey of a particular age or size (Price 1975; King, 2002). Because prevalence of disease commonly varies across age and size classes, this selective predation based on age and size may result in selective predation on infected or uninfected prey (Dobson 1988). Additionally, infected prey in some systems may be preferentially predated if infection increases encounter, detection, or capture rate (e.g., Duffy and Hall 2008; Genovart et al. 2010). While cases of selective predation on infected individuals have been widely investigated (reviewed in Lopez and Duffy 2021; Gutierrez, Minchella, and Bernal 2022), selective predation of healthy individuals has received relatively little attention. There is, however, substantial evidence that some predators avoid infected prey, preferentially attacking uninfected individuals (reviewed in Gutierrez, Minchella, and Bernal 2022; Richards, Drake, and Ezenwa 2022). Here we focus on direct selective predation of predators for infected or uninfected individuals within a population.

Although predators will likely reduce population density in a similar manner regardless of their preference for infected or uninfected individuals, the short‐ and long‐term effects of selective predation on disease prevalence are predicted to be quite different depending on the predator preference (e.g., Hall, Duffy, and Cáceres 2005; Duffy and Hall 2008; Vitale and Best 2019). For example, selective predation upon infected individuals is predicted to decrease infection prevalence in the short term (i.e., the “healthy herds hypothesis;” Hudson, Dobson, and Newborn 1992; Packer et al. 2003), while selective predation on uninfected individuals may increase infection prevalence. These predictions assume that predators remove parasites from the community when they consume infected prey, which may not occur in instances of “sloppy predation,” where predation on infected individuals can aid in the spread of a parasite (Cáceres, Knight, and Hall 2009). Additionally, selective predation on infected individuals is predicted to intensify the coevolutionary arms race between hosts and parasites. In contrast, preferential consumption of healthy individuals is predicted to dampen the reciprocal selective pressures between hosts and parasites, slowing the rate of coevolution through time (Gutierrez, Minchella, and Bernal 2022). Thus, understanding how these divergent strategies of prey selectivity by predators affect host–parasite interactions is essential for predicting changes to disease risk and the fitness payoffs for both hosts and their parasites.

Here, we used a host (*Daphnia dentifera*, hereafter “the host”)‐parasite (*Metschnikowia bicuspidata*, hereafter “the parasite”) system to artificially manipulate selective predation. We documented host population abundance and infection over multiple generations to explore the ecological effects of selective predation for either infected or uninfected individuals. We also measured the resulting host susceptibility across populations and predation treatments to assess the evolutionary consequences of selective predation. Understanding both the ecological and evolutionary implications of selective predation will improve our understanding of the short‐ and long‐term effects of predation on disease dynamics in natural systems.

## 2 Methods and Materials

### 2.1 Study System

The host is a dominant zooplankton and non‐selective grazer in many freshwater lakes in North America (Tessier and Woodruff 2002). The host has a cyclically parthenogenic life cycle where, under ideal conditions in the laboratory (e.g., ample food, space, and light), populations often consist of only females. Males are generally produced in the laboratory when conditions involve crowding, lack of food, and low light (summarized in Ebert 2005). In many population‐level experiments with the host in the laboratory, the proportion of males is very low (< 5% of the population; Searle et al. 2016, Blackwood et al. 2024). The parasite is transmitted horizontally and shows limited genetic variation (Searle et al. 2015). While filter‐feeding for food, hosts can ingest fungal spores and become infected with the parasite (Hall et al., 2007). Although hosts within the genus *Daphnia* can become infected with a variety of parasite taxa (Ebert 2005), we focused on this parasite because it is common within the natural range of the host (Duffy and Hall 2008). Infection can be identified visually in hosts after infection intensifies 9–10 days post‐infection using a stereomicroscope; infection turns the normally transparent hosts opaque (Duffy and Hall 2008; Stewart Merrill and Cáceres 2018). This change in transparency also makes infected hosts more likely to be predated upon by visual predators, including bluegill sunfish ( *Lepomis macrochirus* ; Duffy and Hall 2008). Once visibly infected, hosts are unable to recover from parasite infection, which results in reduced fecundity and shortened life span (Ebert, Lipsitch, and Mangin 2000). After host death, fungal spores are released into the water column (Ebert and Weisser 1997). Given this natural history, transmission is expected to increase with higher host density and higher density of free‐living fungal spores (Anderson and May 1981; Searle et al. 2016). The likelihood that an individual becomes infected with the parasite also varies across host genotypes (Searle et al. 2015), and body size of the host is positively correlated with the likelihood a host becomes infected (Bertram et al. 2013; Stewart Merrill et al. 2019).

### 2.2 Population Experiment

The experiment had three treatments with different predation pressures on the population: (1) selective predation on healthy hosts, where uninfected individuals were removed; (2) selective predation on infected hosts, where infected individuals were removed; and (3) a control group with random predation, where both infected and uninfected hosts were removed. Each treatment consisted of 10 replicate host populations in microcosms, for a total of 30 populations or experimental units. While the microcosms were sampled weekly for a total of 10 weeks (approximately eight or nine asexual generations in these conditions), predation pressure was applied only during the last 6 weeks of the experiment.

We initiated host populations with 75 individuals from 15 different clones (Table S1), five individuals of each clone. The populations were 1 L beakers filled with 800 mL of well water at 20°C with a 16:8 light: dark cycle. Each day we added 2.0 × 10^7^ cells of the alga, *Ankistrodesmus falcatus* as food for the hosts. We conducted a partial water change each week where we first homogenized each beaker via vigorous stirring and then replaced 300 mL with clean well water (a 37.5% water change). Additionally, during water changes, we categorized each individual in a 100 mL subsample of the population based on age (adult or juvenile), sex (male or female), and infection status (infected or uninfected) by placing each individual from the subsample under a stereomicroscope (Olympus SZX16, 0.7–11.5× with darkfield).

After a one‐week acclimation period, we added 1.2 × 10^5^ spores of the parasite to each population to create a concentration of 150 spores mL^−1^ in each beaker. The microcosms experienced no predation pressure during the first 4 weeks of the experiment to allow the host and parasite populations to grow and establish. Thereafter, all individuals in the 300 mL of water removed for weekly water changes were visually identified under a stereomicroscope and removed as specified for each treatment. For selective predation on healthy individuals, all uninfected individuals were removed; for selective predation on infected individuals, all visibly infected individuals were removed; and for our random treatment, all individuals were removed. The remaining hosts from the 300 mL sample were placed back into their respective beakers.

We calculated the host population abundance, abundance of infected hosts, infection prevalence, and the proportion of juveniles in the host population for each microcosm over weeks 4–10 (i.e., after the predation treatments began) and compared these responses across treatments. To control for variations in population growth and infection among replicates, a single “integrated” value was calculated using the trapezoidal area under the curve per replicate for each response variable (following Civitello et al. 2013; Searle et al. 2016). We then compared the integrated population abundance, integrated abundance of infected hosts, integrated infection prevalence, and integrated proportion of juveniles across treatments using an ANOVA with predation treatment as the explanatory variable. Significant interactions were followed by Tukey's honestly significant difference (HSD) tests.

### 2.3 Population Susceptibility

To investigate the effects of predation on selection for host disease susceptibility, we measured infection at the end of the 10‐week microcosm experiment. Here, we use the term “susceptibility” to indicate the likelihood that an individual becomes visibly infected with the parasite given a standard dose. Based on this metric, highly susceptible individuals are more likely to become infected, and individuals with low susceptibility are less likely to become infected. Immediately after our final, week 10 population census, we collected 20 individuals from each microcosm and isolated them into individual 50 mL beakers. To control for maternal and environmental effects, we reared these individual lines for three generations (Coldsnow et al. 2017) and then exposed one individual from each maternal line to the parasite individually in 100 mL of water and 150 parasite spores mL^−1^. Due to mortality during the maternal lines, the number of animals exposed to the parasites ranged from 2 to 18 (average: 10.9 ± 4.6) from each microcosm. Infection status of each individual was assessed 10 days after parasite exposure, where each individual was classified as infected or uninfected using a stereomicroscope. The proportion of infected individuals for each original microcosm was then calculated to estimate the average susceptibility of the population in each microcosm. We also measured the length of up to three additional individuals from each of the maternal lines after rearing them to age 7–8 days old. We measured the length from the middle of the eye to the base of the tail using a stereomicroscope and cellSens imaging software. Infection prevalence was compared across predation treatments using a binomial generalized linear model, taking into account the different sample sizes across replicates by binding the number of infected and uninfected animals together for each replicate as our predictor variable. Length was compared across treatments using an ANOVA after averaging the size of individuals within a clone and then beaker (i.e., one value for each replicate). Significant results were followed with pairwise comparisons using the same models.

## 3 Results

**FIGURE 1:**
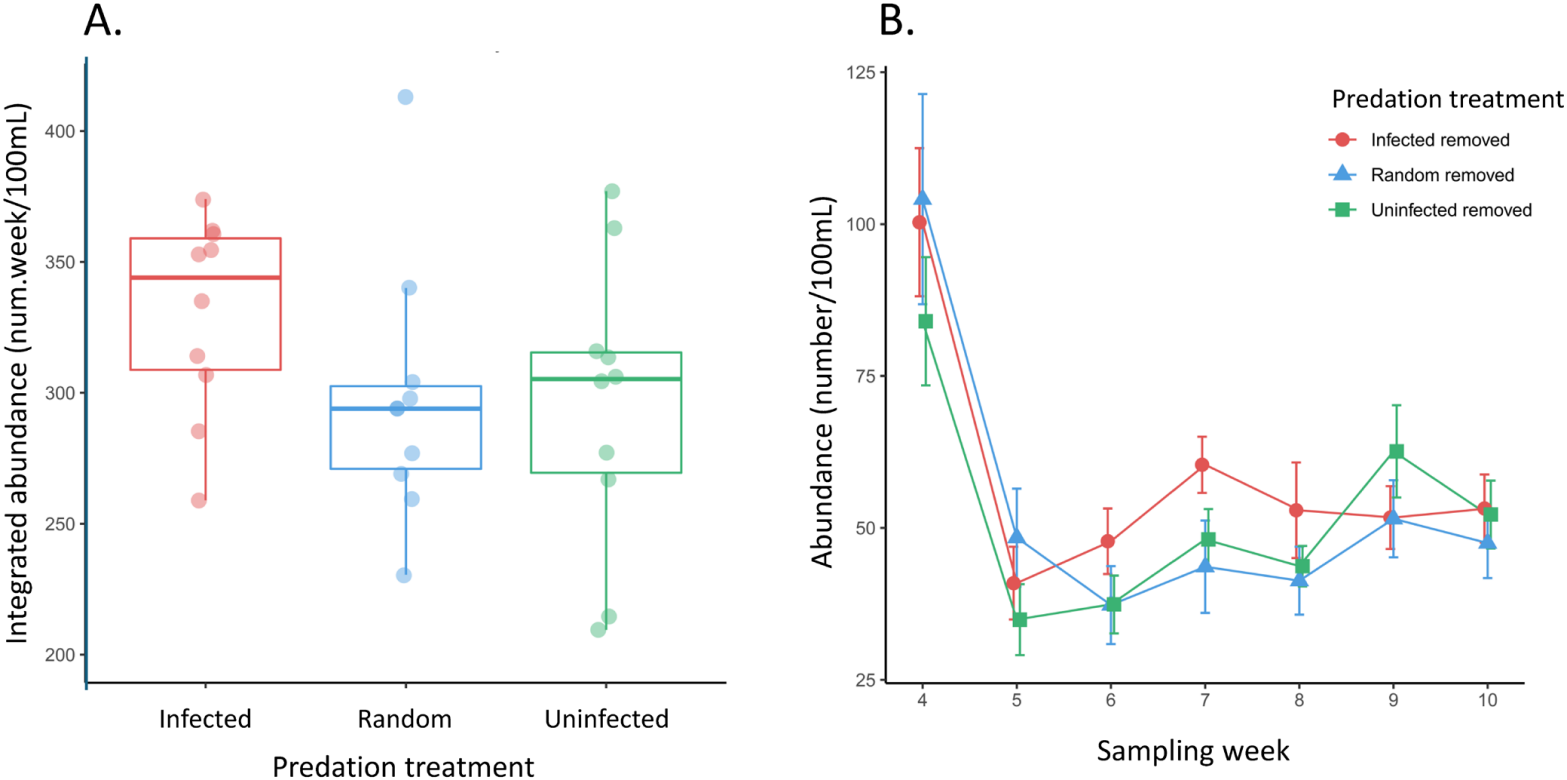
Population abundance across predation treatments in the microcosms. Selective predation was applied by removing infected or uninfected individuals, while the random predation removed both classes and acted as a control treatment. (A) Integrated abundance from the weekly subsamples did not statistically differ across treatments. Boxplots show median and interquartile range with lines showing the range without outliers and individual points showing each replicate (*n* = 10 for each treatment). (B) The time series of average population abundance in the subsample across treatments (±SE) is shown for weeks 4–10 when the predation treatments were applied.

**FIGURE 2:**
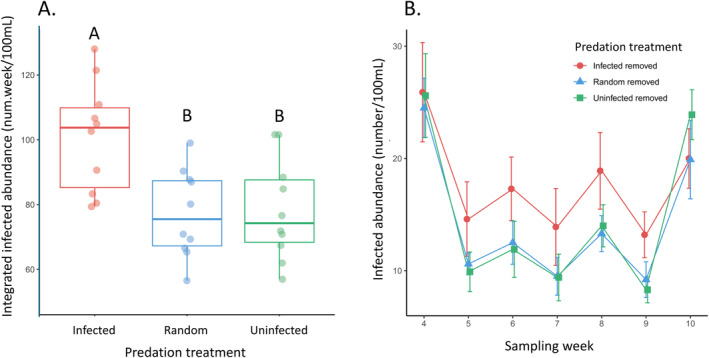
The abundance of infected individuals across predation treatments in the microcosms. Selective predation was applied by removing infected or uninfected individuals, while the random predation removed both classes and acted as a control treatment. (A) Integrated infected abundance from the weekly subsamples was higher in the treatment where infected individuals were removed compared to the two other treatments. Boxplots show median and interquartile range with lines showing the range and individual points showing each replicate (*n* = 10 for each treatment). Treatments that share letters are not statistically different from one another. (B) The time series of average infected abundance in the subsample across treatments (±SE) is shown for weeks 4–10 when the predation treatments were applied.

**FIGURE 3:**
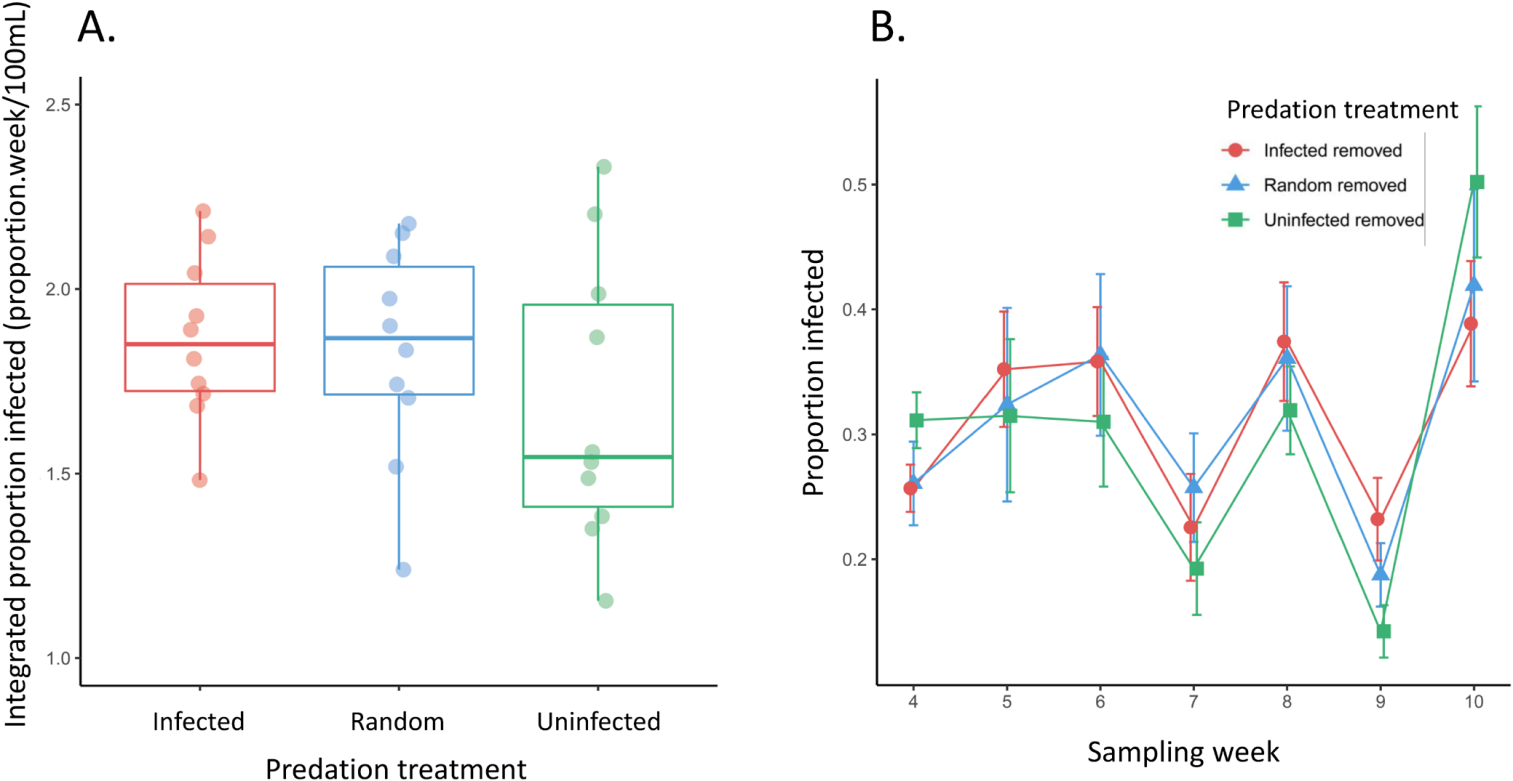
The proportion of infected individuals across predation treatments in the microcosms. Selective predation was applied by removing infected or uninfected individuals, while the random predation removed both classes and acted as a control treatment. (A) Integrated proportion infected from the weekly subsamples did not statistically differ across treatments. Boxplots show median and interquartile range with lines showing the range and individual points showing each replicate (*n* = 10 for each treatment). (B) The time series of average infection prevalence in the subsample across treatments (±SE) is shown for weeks 4–10 when the predation treatments were applied.

Across all weeks, treatments, and replicates, we counted 19,234 individuals. Only 104 were male (0.54% of the population). There were no differences in integrated population abundance across predation treatments (*F*_2,27_ = 1.99, *p* = 0.156; Figure 1). However, we found differences across treatments in integrated infected host abundance; populations where infected individuals were removed had higher integrated infected host abundance compared to populations in the other two treatments (*F*_2,27_ = 7.50, *p* = 0.003; Tukey *p* < 0.01 comparing the treatment with infected individuals removed to the two other treatments; Tukey *p* = 0.990 comparing treatments with random versus uninfected hosts removed; Figure 2). However, there were no differences in the integrated proportion of infected individuals (*F*_2,27_ = 0.95, *p* = 0.400; Figure 3), integrated juvenile proportion (*F*_2,27_ = 2.93, *p* = 0.071; Figure S1), or body length (*F*_2,27_ = 0.14, *p* = 0.874; Figure S2) across treatments.

**FIGURE 4:**
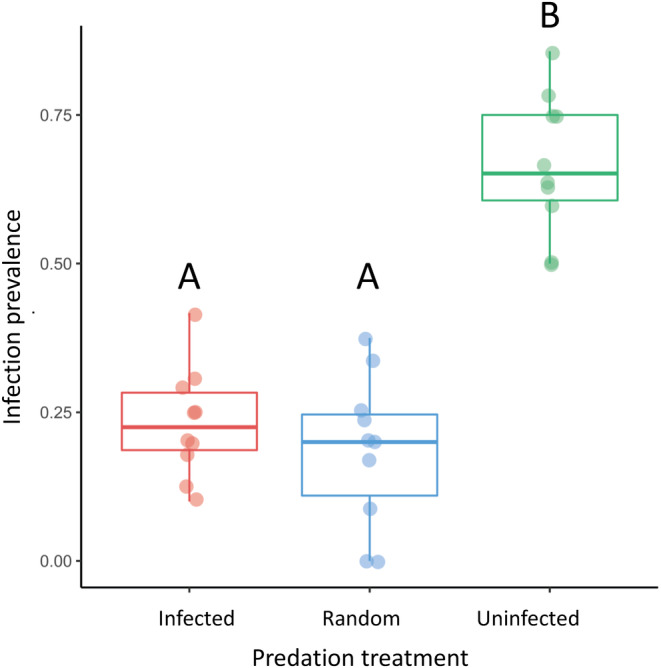
Infection results from the population susceptibility experiment. Boxplots show median and interquartile range with lines showing the range and individual points showing the infection prevalence for each replicate (*n* = 10 for each treatment). Treatments that share the same letter do not statistically differ. Post‐microcosm infection prevalence was highest in the treatment with uninfected individuals removed compared to the two other predation treatments.

In our population‐level test of susceptibility, we found differences across treatments (*X* ^2^(2) = 20.99, *p* < 0.001; Figure 4) where populations with predation on uninfected individuals had higher susceptibility than populations with random predation or predation on infected individuals (*p* < 0.01 for both comparisons), while the random and infected removed treatments did not differ from each other (*X* ^2^(1) = 0.01, *p* = 0.914; Figure 4).

## 4 Discussion

The establishment and persistence of a parasite in a host population and the resulting disease dynamics can depend on other members of a host's community. Previous theoretical studies have suggested that predation can reduce parasitism, “keeping herds healthy” by reducing host densities and culling infected hosts (Packer et al. 2003). Extensive research on predation and host–parasite interactions has highlighted the highly variable effects predators can have on prey and their parasites (Wilson, Fenton, and Tompkins 2019; Lopez and Duffy 2021; Richards, Drake, and Ezenwa 2022). Although numerous studies have illustrated different mechanisms by which predators may increase disease within prey populations, identifying the mechanisms, circumstances, and their subsequent outcomes remains difficult (Holt and Roy, 2007; Stephenson, van Oosterhout, and Cable 2015; Buss and Hua 2018; Richards, Drake, and Ezenwa 2022).

Several previous studies on *Daphnia*‐parasite systems have shown evidence of the strong effects predators may have on disease prevalence in host populations (e.g., Johnson et al. 2006; Duffy 2007). Hosts infected with the parasite in this system become more opaque, making them more conspicuous and therefore more vulnerable to visual predators, an example of selective predation for infected individuals (Duffy and Hall 2008). Accordingly, because we completely removed predated individuals from the population (i.e., not inducing “sloppy predation,” Cáceres, Knight, and Hall 2009), we predicted that populations with selective predation on infected individuals would have the lowest infection. Contrary to this prediction, we found the highest abundance of infected individuals in treatments where selective predation occurred on infected individuals (Figure 2). There are several potential explanations for this unexpected result. First, in some systems, individual traits of hosts (e.g., behavior; Curtis 2014) can change depending on the abundance of parasites. Thus, the hosts in our experiment may have altered their phenotype or behavior in response to the different predation treatments, in a manner that changed their likelihood of becoming infected. Second, although this parasite is generally expected to transmit in a density‐dependent manner (Searle et al. 2016), the likelihood a host becomes infected is not always positively correlated with parasite exposure, and U‐shaped dose–response curves (i.e., hermetic effect) that have been observed in other systems may occur in some scenarios in this system (Bauer et al. 2024). Finally, because we only applied predation pressure on a sub‐sample of the population, without causing detectable differences in abundance across treatments (Figure 1), it is possible that our predation regime was not strong enough to elicit the predicted patterns. However, a weak predation regime seems unlikely given that predation pressure was applied to nearly 40% of the population, this level of predation differences in susceptibility (Figure 4), and a large effect of predation has been seen in similar microcosm studies (Richards, Drake, and Ezenwa 2022). Additionally, it is possible that our treatments induced selection on parasite virulence, but we do not expect that parasite evolution was a strong driver of our observed patterns because the parasite exhibits very low genetic diversity (Searle et al. 2015). Future work in this system could investigate how the intensity of the predation treatments and the genetic diversity of the parasite influence eco‐evolutionary dynamics.

In our system, infection in the host can be visually identified approximately 9–10 days after parasite exposure, when asci of the parasite fill the host's hemolymph (Stewart Merrill and Cáceres 2018). When we implemented selective predation on infected individuals, we likely failed to remove infected animals that had early‐stage infections that were not visible. Conversely, in our treatments with selective predation on uninfected individuals, we likely removed some individuals who had non‐visible, early‐stage infections. These predation regimes represent what would be expected to occur with a visually oriented predator but may have dampened the effects of selective predation on the hosts. However, the number of parasite spores found in early‐stage infections (i.e., before infections can be seen) is very low (Auld et al. 2014; Auld, Wilson, and Little 2014), such that the impact of mischaracterizing these infections is likely to be small.

Often when investigating the impacts of selective predation, the indirect effects on prey population dynamics can be overlooked. For instance, removing a large portion of a particular class within a population can have cascading effects on other groups. Rates of growth and maturation could be affected for other classes, influencing disease dynamics. For example, by removing larger‐sized individuals, resources may become more readily available to smaller or younger prey. Such an increase in resources could result in accelerated growth and higher reproduction rates (Abrams and Rowe 1996; Relyea 2007). Because susceptibility to disease often changes with age (Ben‐Ami 2019), changes in developmental rates could alter population‐level disease dynamics. In addition to potential ecological changes in population dynamics in response to a predator, tradeoffs between predation and infection risk could lead to rapid evolution that may ultimately promote disease (Buss and Hua 2018). While we did not detect differences in age or size across predation treatments (Figures S1 and S2), it is possible that other aspects of the populations varied across our treatments in ways that impacted infection and abundance.

At the end of the experiment, susceptibility was highest in the treatments with predation upon uninfected hosts (Figure 4). This result may have occurred due to the high cost of parasite resistance in this treatment, where uninfected individuals were less likely to survive and reproduce than infected individuals. While resistant alleles are generally expected to be beneficial during times of high parasite infection, they can come with inherent costs (Hall et al. 2010), which may have been exacerbated when predation was also higher for individuals with resistant alleles. Although we did not observe the complementary result of lower susceptibility in treatments where infected individuals were predated upon compared to the random treatment, this pattern might be expected to occur in some systems where the costs of infection and predation upon infected individuals are high. In all, our susceptibility results indicate that selective predation may have long‐term effects on disease mediated through selection on hosts.

The effects of predation on prey disease dynamics have important public health and conservation implications (Packer et al. 2003; Ostfeld and Holt 2004). However, it is critical to consider the possible interactions between predation strategies and parasites when making predictions about disease dynamics. We found effects of selective predation on both short‐term infected host abundance and selection for parasite susceptibility. Together, our results highlight the importance of understanding host–parasite interactions in a community context, from both an ecological and evolutionary perspective.

## Author Contributions

**Stephanie O. Gutierrez:** conceptualization (equal), data curation (lead), formal analysis (lead), investigation (lead), methodology (equal), writing – original draft (lead). **Ximena E. Bernal:** conceptualization (equal), methodology (equal), supervision (equal), writing – review and editing (equal). **Catherine L. Searle:** conceptualization (equal), formal analysis (supporting), methodology (equal), resources (lead), writing – review and editing (equal).

## Conflicts of Interest

The authors declare no conflicts of interest.

## Supporting information


Appendix S1.

## Data Availability

Data from this project are available at: https://doi.org/10.5061/dryad.qjq2bvqrt.
