# Supplementary material for: Impacts of Selective Predation on Infection Prevalence and Host Susceptibility
Source: Ecol Evol. 2025 Jan 21;15(1):e70778. doi: 10.1002/ece3.70778 (PMC11747352; doi:10.1002/ece3.70778)
Supplement: Supplementary file 1 — Appendix S1. [file ECE3-15-e70778-s001.pdf]

Supplemental Materials for "Impacts of selective predation on infection prevalence and host susceptibility"

Authors: Stephanie O. Gutierrez, Ximena E. Bernal, Catherine L. Searle

Journal: *Ecology and Evolution*

Table S1: List of clones used in the experiment and the lake where they were collected.

Clone name	Lake of collection	U.S. State of collection
Standard	Unknown lake in Barry County	Michigan
Mid 37	Midland	Indiana
Wd 50	Walsh	Michigan
Gam 303	Gambill	Indiana
Beav 314	Beaver Dam	Indiana
Bd 16	Bishop	Michigan
Good 301	Goodman	Indiana
FFSW 10	Fairfield Southwest	Indiana
FFSW 6	Fairfield Southwest	Indiana
FFSW 5	Fairfield Southwest	Indiana
FFSW 9	Fairfield Southwest	Indiana
FFSW 12	Fairfield Southwest	Indiana
FFNE 14	Fairfield Northeast	Indiana
FFNE 6	Fairfield Northeast	Indiana
FFNE 15	Fairfield Northeast	Indiana

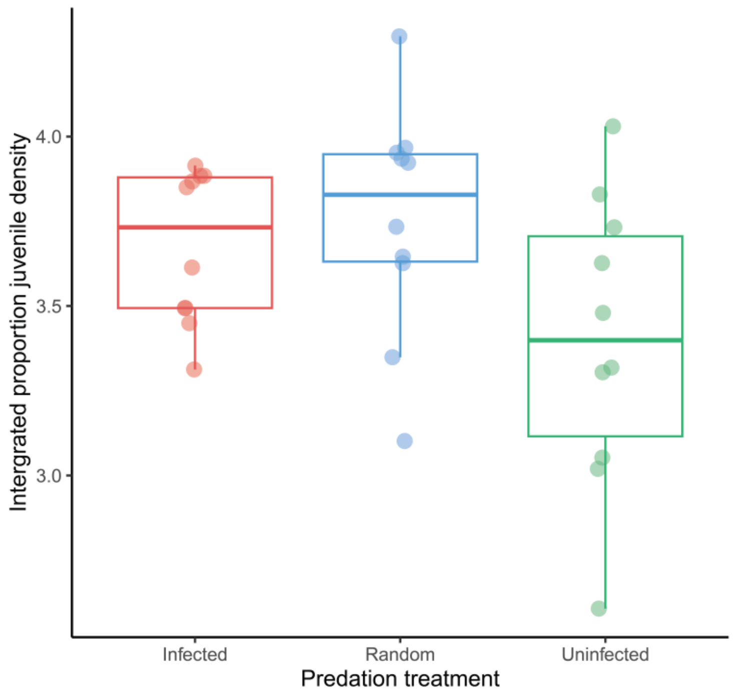

Supplemental Figure 1: The integrated value of the proportion of juveniles in the microcosm experiment. Boxplots show median and interquartile range with lines showing the range without outliers, and individual points showing the integrated values for each replicate (n = 10 for each treatment). There were no statistically significant differences in the proportion of juveniles across treatments.

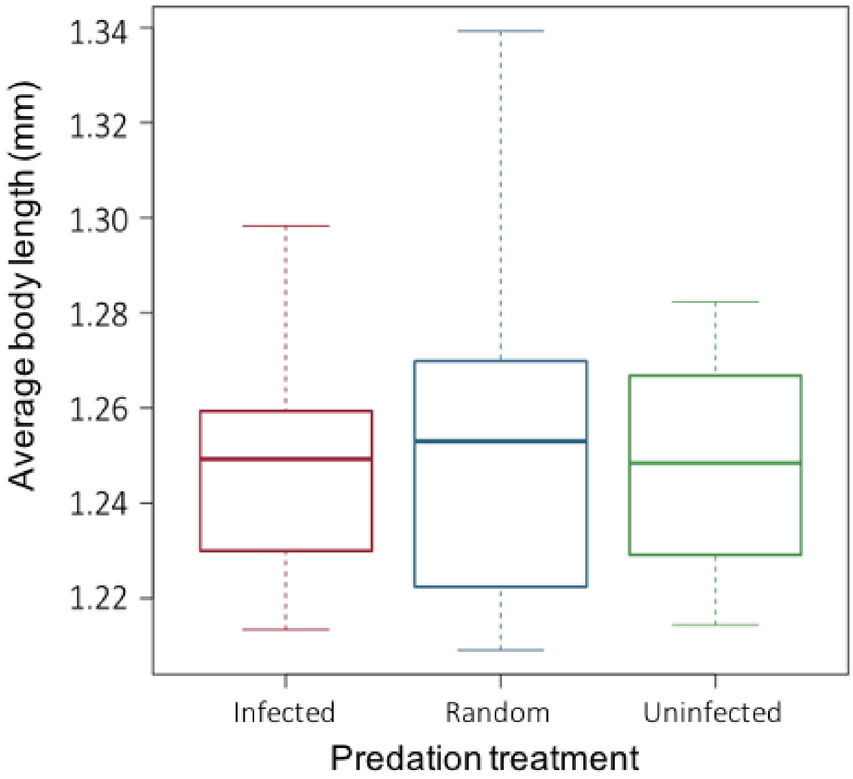

Supplemental Figure 2: Average body length from the post-microcosm experiment across replicates and predation treatments. Boxplots show median and interquartile range with lines showing the range. There were no statistically significant differences in body size across treatment.
